# Supplementary material for: Preserved neural dynamics across animals performing similar behaviour
Source: Nature. 2023 Nov 8;623(7988):765–71. doi: 10.1038/s41586-023-06714-0 (PMC10665198; doi:10.1038/s41586-023-06714-0)
Supplement: Supplementary file 1 — Reporting Summary [file 41586_2023_6714_MOESM1_ESM.pdf]

nature portfolio

Corresponding author(s): Juan A. Gallego
Matthew G. Perich

Last updated by author(s): 2023.08.23

Reporting Summary

Nature Portfolio wishes to improve the reproducibility of the work that we publish. This form provides structure for consistency and transparency in reporting. For further information on Nature Portfolio policies, see our Editorial Policies and the Editorial Policy Checklist.

Statistics

For all statistical analyses, confirm that the following items are present in the figure legend, table legend, main text, or Methods section.

n/a	Confirmed	
☐	☒	The exact sample size (*n*) for each experimental group/condition, given as a discrete number and unit of measurement
☒	☐	A statement on whether measurements were taken from distinct samples or whether the same sample was measured repeatedly
☐	☒	The statistical test(s) used AND whether they are one- or two-sided<br>*Only common tests should be described solely by name; describe more complex techniques in the Methods section.*
☒	☐	A description of all covariates tested
☒	☐	A description of any assumptions or corrections, such as tests of normality and adjustment for multiple comparisons
☐	☒	A full description of the statistical parameters including central tendency (e.g. means) or other basic estimates (e.g. regression coefficient) AND variation (e.g. standard deviation) or associated estimates of uncertainty (e.g. confidence intervals)
☐	☒	For null hypothesis testing, the test statistic (e.g. *F*, *t*, *r*) with confidence intervals, effect sizes, degrees of freedom and *P* value noted<br>*Give P values as exact values whenever suitable.*
☒	☐	For Bayesian analysis, information on the choice of priors and Markov chain Monte Carlo settings
☒	☐	For hierarchical and complex designs, identification of the appropriate level for tests and full reporting of outcomes
☒	☐	Estimates of effect sizes (e.g. Cohen's *d*, Pearson's *r*), indicating how they were calculated

Our web collection on statistics for biologists contains articles on many of the points above.

Software and code

Policy information about availability of computer code

Data collection	Monkey dataset: Neural data was acquired using software from Blackrock Microsystems (Salt Lake City, USA); behavioural data was acquired using custom Matlab code (The Mathworks, Inc.). Mouse dataset: Neural data was acquired using the SpikeGLX software v. 3.0 (HHMI/Janelia Research Campus, Ashburn USA), preprocessed using Kilosort 2.0 (https://github.com/MouseLand/Kilosort), and manually curated using Phy (https://github.com/cortex-lab/phy}; behavioural data was estimated from videos using the video annotation tool JAABA (Kabra et al, Nature Methods, 2013); joystick operations were controlled using a custom Python package: https://github.com/janelia-pypi/mouse_joystick_interface_python.
Data analysis	All data analysis was performed in Python using open source packages such as numpy, matplotlib, sci-kit, scipy and pandas. All the analysis code is publicly available at https: //github.com/BeNeuroLab/2022-preserved-dynamics

For manuscripts utilizing custom algorithms or software that are central to the research but not yet described in published literature, software must be made available to editors and reviewers. We strongly encourage code deposition in a community repository (e.g. GitHub). See the Nature Portfolio guidelines for submitting code & software for further information.

Data

Policy information about availability of data

All manuscripts must include a data availability statement. This statement should provide the following information, where applicable:

- Accession codes, unique identifiers, or web links for publicly available datasets
- A description of any restrictions on data availability
- For clinical datasets or third party data, please ensure that the statement adheres to our policy

The majority of the monkey datasets are publicly available on Dryad (https://datadryad.org/stash/dataset/doi:10.5061/dryad.xd2547dkt) and CRCNS (http://dx.doi.org/10.6080/K0FT8J72). The remaining monkey datasets and the mouse datasets will be made available upon reasonable request (these data have yet not been appropriately curated for public distribution).

Research involving human participants, their data, or biological material

Policy information about studies with human participants or human data. See also policy information about sex, gender (identity/presentation), and sexual orientation and race, ethnicity and racism.

Reporting on sex and gender	N/A
Reporting on race, ethnicity, or other socially relevant groupings	N/A
Population characteristics	N/A
Recruitment	N/A
Ethics oversight	N7A

Note that full information on the approval of the study protocol must also be provided in the manuscript.

Field-specific reporting

Please select the one below that is the best fit for your research. If you are not sure, read the appropriate sections before making your selection.

☒ Life sciences ☐ Behavioural & social sciences ☐ Ecological, evolutionary & environmental sciences

For a reference copy of the document with all sections, see nature.com/documents/nr-reporting-summary-flat.pdf

Life sciences study design

All studies must disclose on these points even when the disclosure is negative.

Sample size	We analyzed data from four monkeys (including two different behavioral tasks) and four mice to show reproducibility. Overall, our results include as many as 32 sessions across the two monkey datasets, and six sessions for the mice dataset. This sample size is larger than most studies in the field —in non-human primate research, a few sessions across n = 2 individuals is considered to be sufficient. In both species, we simultaneously recorded from enough neurons (or neural units in the case of one monkey), to reliably estimate the neural population latent dynamics that our results are based upon (see, e.g., Churchland et al Neuron 2010, Gallego et al Nature Comms, 2018, Sadtler et al Nature 2014, Trautman et al Neuron 2019).
Data exclusions	No data were excluded from analysis.
Replication	We replicated the core findings across two species (mice and non-human primates), four behaviors (a centre-out reaching task, a sequential reaching task, and a reach, grasp and pull task, along with during covert movement planning), and two brain regions (motor cortex and dorsolateral striatum). Experiments on each species were performed independently in two different laboratories, and by different scientists. The mice experiments were done in a single cohort, whereas the monkey data was collected in two sets of experiments (one for the centre-out task, another for the random reaching task), each spanning two years. Overall, our neural recordings and behavioural data are in good agreement with related published studies. All attempts at replication were successful.
Randomization	Covariates were not experimentally controlled so randomization was not necessary for our study (because all the animals were assigned to the same group).
Blinding	Blinding was not necessary for our study as we were not assessing the effect of any manipulation or comparing across conditions.

Reporting for specific materials, systems and methods

We require information from authors about some types of materials, experimental systems and methods used in many studies. Here, indicate whether each material, system or method listed is relevant to your study. If you are not sure if a list item applies to your research, read the appropriate section before selecting a response.

Materials & experimental systems

n/a	Involved in the study
☒	☐ Antibodies
☒	☐ Eukaryotic cell lines
☒	☐ Palaeontology and archaeology
☐	☒ Animals and other organisms
☒	☐ Clinical data
☒	☐ Dual use research of concern
☒	☐ Plants

Methods

n/a	Involved in the study
☒	☐ ChIP-seq
☒	☐ Flow cytometry
☒	☐ MRI-based neuroimaging

Animals and other research organisms

Policy information about studies involving animals; ARRIVE guidelines recommended for reporting animal research, and Sex and Gender in Research

Laboratory animals	We recorded from four non-human primates (three macaca mulatta, one macaca fascicularis; all male, aged 6-10 years) and four mice (C57BL/6; aged 8-16 weeks). Mice were maintained on a 12-12 h (8am-8pm) light/dark cycle and recordings were made between 9am and 3pm. The holding room temperature was maintained at 21+/-1°C with a relative humidity of 30% to 70%.
Wild animals	No wild animals were used in this study.
Reporting on sex	Sex was not considered in this study.
Field-collected samples	This study did not involve samples collected from the field.
Ethics oversight	All surgical and experimental procedures for the monkey dataset were approved by the Institutional Animal Care and Use Committee of Northwestern University under protocol \#IS00000367. All surgical and experimental procedures for the mouse dataset were approved by the Institutional Animal Care and Use Committee of Janelia Research Campus.

Note that full information on the approval of the study protocol must also be provided in the manuscript.
